# ﻿Taxonomy of *Landrevus* species group of *Velarifictorus* Randell, 1964 (Orthoptera, Gryllidae, Gryllinae) with one new species and morphological diversity of *Velarifictorusflavifrons* Chopard, 1966

**DOI:** 10.3897/zookeys.1084.77096

**Published:** 2022-01-28

**Authors:** Yan-Na Zheng, Xin-Ru Cai, Li-Bin Ma

**Affiliations:** 1 College of Life Sciences, Shaanxi Normal University, Xi’an, 710119, China Shaanxi Normal University Xi'an China

**Keywords:** Calling song, cricket, Grylloidea, Modicogryllini, new species group

## Abstract

The *Landrevus* species group includes four *Velarifictorus* species that are related to Landrevinae crickets (*Velarifictoruselephas* Gorochov, 1992, *Velarifictorusbubalus* Gorochov, 1992, *Velarifictorusgradifrons* Ingrisch, 1998, and *Velarifictoruslandrevus* Ma, Qiao & Zhang, 2019). A new species of the group is discovered in the Yunnan Province of China, and it is described and illustrated here. *Velarifictorusyunnanensis* Liu & Yin, 1993 is recognized as a junior synonym of *Velarifictorusflavifrons* Chopard, 1966. The morphological variety of *V.flavifrons* ectoparamere is documented and studied.

## ﻿Introduction

There are 110 species of *Velarifictorus* Randell, 1964 (Orthoptera, Gryllidae, Gryllinae: Modicogryllini) in the world (Cigliano et al. 2021). Four members of the genus are closely related and look remarkably like crickets of the subfamily Landrevinae; they are dark or chocolate brown, the tegmina are short, and they have a similar type of epiphallus. *Velarifictoruselephas* Gorochov, 1992, *Velarifictorusbubalus* Gorochov, 1992, *Velarifictorusgradifrons* Ingrisch, 1998 and *Velarifictoruslandrevus* Ma, Qiao & Zhang, 2019 are the species that we recognize as belonging to the *Landrevus* species group. We identified a fifth species from Yunnan Province, China, which is similar to these, and we describe it as a new species for science.

Among the species of the genus *Velarifictorus*, *Velarifictorusyunnanensis* Liu & Yin, 1993 has been recorded from China. Gorochov (2001) designated it as a junior synonym of *Velarifictorusflavifrons* Chopard, 1966. We compared the illustrations in the original descriptions of both species and conclude that they are not the same. We analyzed many samples from Yunnan Province referred to as either *V.flavifrons* or *V.yunnanensis* to confirm the relationship between the two taxa. We also report on the morphological variation of the male genitalia of *V.flavifrons* and validate the synonymy as stated by Gorochov (2001).

## ﻿Materials and methods

The specimens were preserved in absolute analytical-grade ethanol during fieldwork and then pinned and dry-preserved in the laboratory. Identification of species is mainly based on male morphology. Illustrations of head and genitalia were taken with a ToupCam digital camera and bundled software (ToupTek, Hangzhou, China). Habitats and specimen photographs were obtained using a Keyence VHX-6000 super-high magnification lens zoom 3D microscope (Keyence, Japan). The details of ovipositor were obtained using JEC-6500 lon sputtering instrument (Hitachi, Japan) and TM3030Plus tabletop electron microscopy (Jeol, Japan). Genitalia were prepared by placing dissected genitalia into a solution of alkaline proteinase (0.2g/ml; AOBOX, Beijing, China) with water temperature of 40–50 °C for 48 h.

### ﻿Song recording and analyses

All the specimens were kept singly in a plastic box (diameter 20 mm, height 50 mm) with small holes in the laboratory. We recorded songs overnight by placing a Sony PCM-D50 (Sony, China) recorder near the box, and replayed songs in Raven Lite v. 2.0 (Bioacoustics Research Program, Cornell Lab of Ornithology).

### ﻿Measurements

All specimens were measured using ToupCam digital camera (E3ISPM05000KPA) and bundled software (ToupTek, Hangzhou, China). All measurements are in millimeters (mm).

### ﻿Abbreviations

**Measurements**:

**BL** body length (from head to tip of abdomen);

**HL** head length;

**HW** head width;

**PL** pronotum length;

**PW** pronotum width (maximum width of pronotum);

**FWL** tegmen (forewing) length;

**HFL** hind femur length;

**OL** ovipositor length.

**Male genitalia**:

**R** rami;

**G** guiding rod;

**Ect** ectoparamere;

**M. Ep** medial lobes of epiphallus;

**L. Ep** lateral lobes of epiphallus.

All specimens studied in the article are deposited in the museum of Flora and Fauna of Shaanxi Normal University, Xi’an, China (**SNNU**).

## ﻿Taxonomy

### ﻿Orthoptera, Grylloidea, Gryllidae, Gryllinae, Modicogryllini

#### ﻿Genus Velarifictorus

Taxon classificationAnimaliaOrthopteraGryllidae

Randell, 1964

68AA2624-9CEA-572D-AD70-FB1A4C9EA65A

##### Type species.

*Scapsipedusmicado* Saussure, 1877.

#### ﻿ Velarifictorus zhengi

Taxon classificationAnimaliaOrthopteraGryllidae

Zheng & Ma sp.nov.

4B99771A-3479-51A8-A7AA-2DAF8EDE8FD2

http://zoobank.org/160DF768-6BE9-4845-B8AE-AC1897B84F37

: 郑氏斗蟋 Figures 1 , 2 , 3 , 4 , 8A, D , 9A, D , 10A, D , 13A, D , 14A, B ; Table 1

##### Type material.

***Holotype*.** Male. China: Yunnan, Pu’er, Meizihu Park, 22°74.6'N, 100°97.6'E, Ⅷ–18–2021, Zhixin He, Ning Wang, and Wei Yuan leg. (SUUN); ***Paratypes*.** 4 males and 8 females. China: Yunnan, Pu’er, Meizihu Park, 22°74.6'N, 100°97.6'E, Ⅷ–18–2021, Zhixin He, Ning Wang, and Wei Yuan leg. (SUUN). All specimens were found in leaf litter.

**Figure 1.**
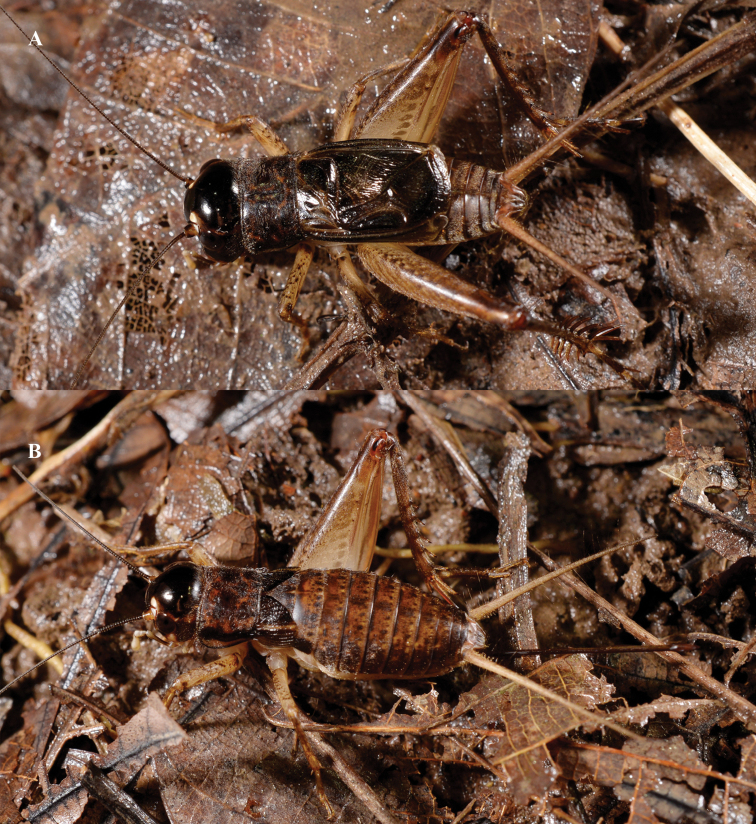
*Velarifictoruszhengi* sp. nov. Habitus (alive) in field **A** male **B** female (photographed by Zhixin He).

##### Measurements.

**Male (*n* = 5)**: BL 15.73±0.14, HL 3.63±0.31, HW 5.47±0.08, PL 3.09±0.03, PW 5.32±0.12, FWL 7.17±0.09, HTL 10.44±0.68; **Female (*n* = 8)**: BL 16.93±0.72, HL 3.11±0.53, HW 4.85±0.13, PL 3.14±0.03, PW 5.00±0.24, FWL 2.50±0.03, HTL 10.94±0.61, OL 13.00±0.09.

**Figure 2.**
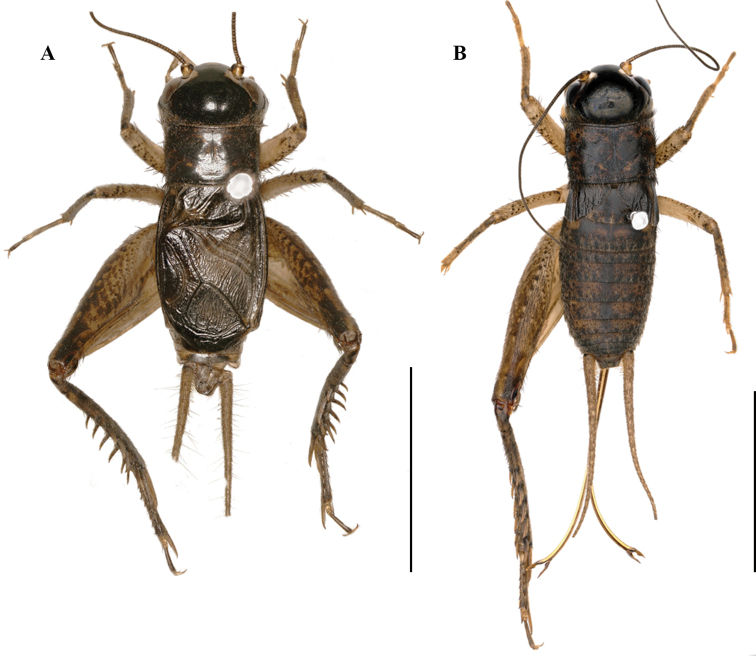
Bodies of *Velarifictoruszhengi* sp. nov. **A** male **B** female. Scale bars: 10 mm.

##### Etymology.

Prof. Zhe-Min Zheng passed away on 16 September 2021. He was a well-known orthopterist in China, and he made outstanding contributions to the taxonomy of Chinese grasshoppers. To honor him, we named this new species after him.

**Figure 3.**
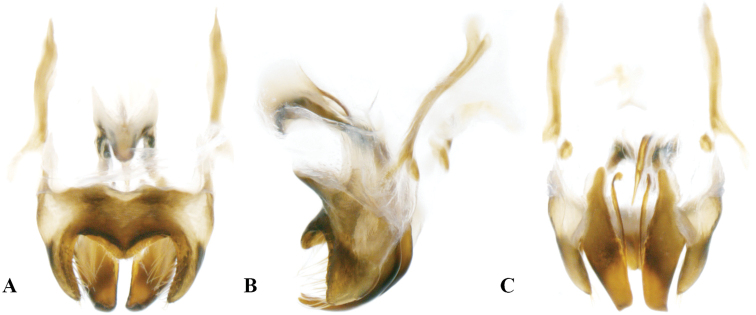
Genitalia of *Velarifictoruszhengi* sp. nov. **A** dorsal view **B** lateral view **C** ventral view.

##### Description.

**Male**: vertex broad and flattened, rather inclined. Occiput bright, slightly convex and somewhat wider than pronotum. Frontal rostrum convex, inclined dorsally and ventrally, and nearly three times wider than antennal scape. Antennal scape shield-like. Ocelli transversely ovoid, almost equal in size. Eyes slightly convex, about 1/4 length of head. Postclypeus shaped as a narrow band, lower margin concave; anteclypeus shaped as a broad shield. Labrum shaped as rhombus, apical margin slightly round. End section of maxillary palpi is about 2/3 length of the third section; end section of labial palpi depressed and widened, almost equal to the total length of all other sections.

**Table 1.** Descriptive statistics of acoustic parameters in calling song samples of *Velarifictoruszhengi* sp. nov.

	*n*	minimum	maximum	mean	std
Chirp interval	26	0.181	3.289	1.735	1.554
Chirp duration	26	0.115	0.191	0.153	0.042
Chirp period	26	1.296	3.480	1.888	1.596
Chirp elements	26	3	5	4	1
Pulse interval	26	0.010	0.022	0.016	0.006
Pulse duration	26	0.031	0.029	0.030	0.001
Pulse period	26	0.041	0.051	0.046	0.007
Peak frequency	26	3.898	6.171	5.035	1.137

The time and frequency parameters are in seconds and kHz respectively. *n*= number of samples analyzed; std= standard deviation.

Pronotum broad and flattened. Median groove of pronotal disc distinct. Posterior margin straight and middle of anterior margin concaved inside, and both margins almost equal in width.

Tegmina reaching eighth abdominal tergite. Oblique veins bifurcated proximally, and each branch of them connected to CuA vein. Three chord veins, connecting to proximal part of mirror by two transverse veins. Mirror shield-like, about twice wider than basal field. Dividing vein absent. Apical field narrow and almost 1/4 length of mirror.

Outer tympanum larger than the inner, inner tympanum oval; outer tympanum elongate-oval. Hind tibiae armed with dorsal spurs (numbered 5:6) almost equal in length; and apical spurs outside three (the dorsal one longest and about two times longer than the dorsal spurs, ventral one about half length of the dorsal, the middle one slightly longer than the dorsal) and inner two (equal in length and about 2/3 length of the dorsal).

##### Genitalia.

Lateral lobes of epiphallus sheet-like, tapering, apically acute, and armed with pilose at the apex. Middle lobe of epiphallus angularity forming an obtuse angle and apically rounded, about 1/3 length of lateral lobes. Ectoparamere stripe-like, tapering, and about 2.5 times longer than epiphallic lateral lobes.

##### Calling song.

**Figure 4.**
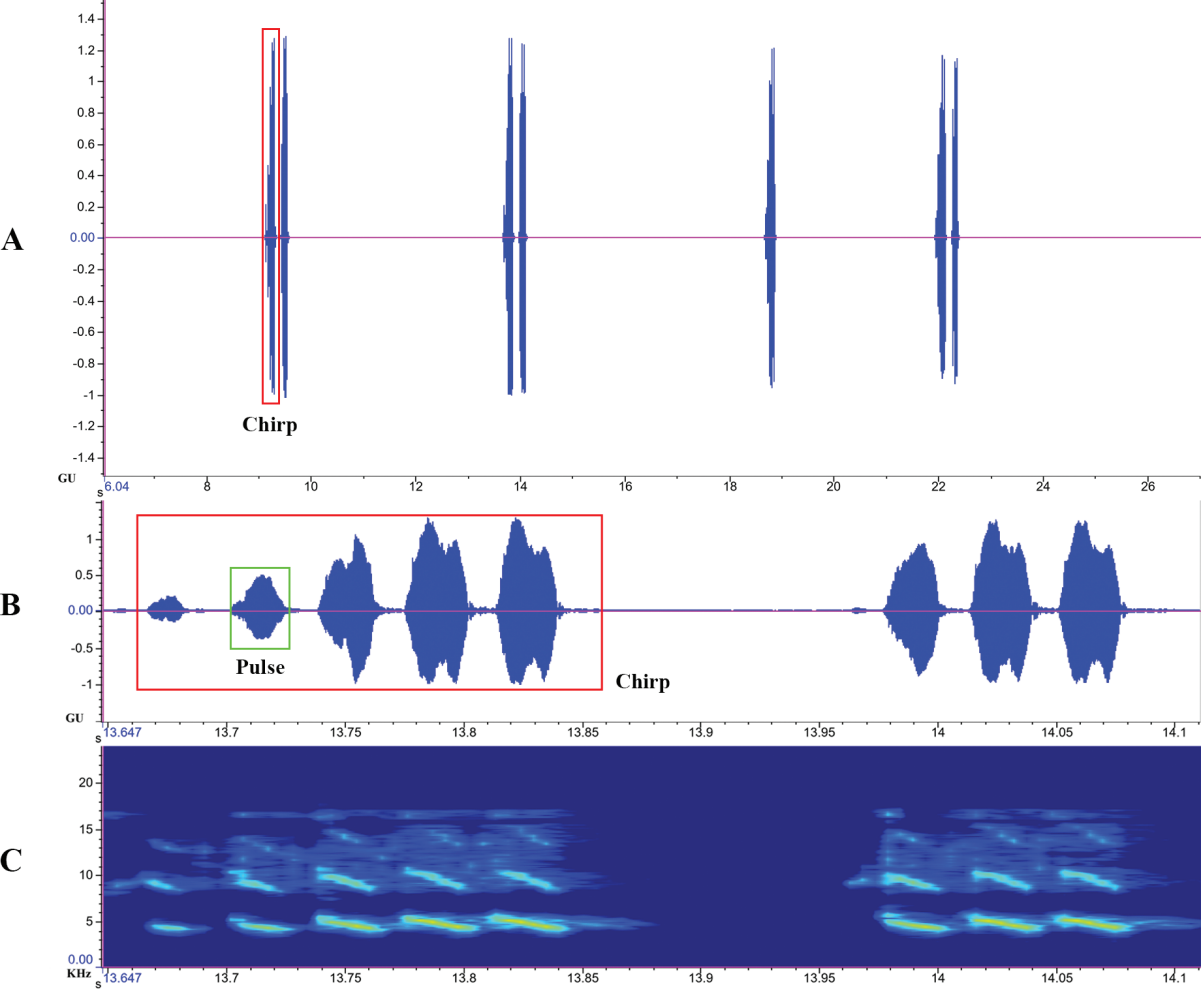
Calling song of *Velarifictoruszhengi* sp. nov. **A** oscillogram of chirp (amplitude versus time) **B** oscillogram of pulse (amplitude versus time) **C** spectrogram (frequency versus time).

Chirps lasting from 1.296 to 3.480 s (mean 1.888), and their duration vary from 0.115 to 0.191 s (mean 0.153). Each chirp equally contains four pulses (Fig. 4B). The peak frequency is between 3.898 and 6.171 kHz (mean 5.035), and the average temperature is 28.0 °C. In terms of spectro-temporal variation, its frequency modulation is inclined downward, which is obvious in the second harmonic.

**Female.** Resembles the male. Head almost as wide as pronotum. Tegmen extends anteriorly to the second abdominal tergite (Fig. 2B), which is shorter on the inside and progressively grows longer on the outside. Dorsally 3 or 4 longitudinal veins and laterally 6 Sc branches (the outermost of which is basally branched) arm the tegmen (Fig. 13D). Ovipositor smooth and slightly longer than the posterior femur. The dorsal ovipositor valve is hooked and has a sharp apex, whereas the apex of the ventral ovipositor valve is slightly rounded (Fig. 14 A, B).

##### Coloration.

Body dark brown. Ocelli light yellow. Eyes brown; between eyes and anterior margin of pronotum marked with yellowish -brown band. Maxillary reddish brown. Cercus and legs yellowish brown.

##### Remarks.

This species resembles species of the *Landrevus* group in features of the body and genitalia. In *V.gradifrons*, however, the inner tympanum is absent and the apical margin of epiphallic middle lobe is straight. In the new species, both tympana are present and the apical margin of epiphallic middle lobe is curved. The apical field of the tegmina of *V.bubalus* and *V.elephas* are longer than in *V.zhengi* sp. nov., and the apical margins of the epiphallic middle lobe are straighter than the new species. Furthermore, the new species is distinguished from *V.landrevus*, another Chinese species, in morphological and acoustical characters. *Velarifictoruszhengi* sp. nov. bears a longer epiphallic middle lobe, whose apex is partially pentagon-like in dorsal view and possesses more pulses (9–15) per chirp at a lower peak frequency. Finally, the absence of a dividing vein in the new species (observed in all type material) is distinct from all other species of the *Landrevus* group.

#### ﻿ Velarifictorus (Velarifictorus) landrevus

Taxon classificationAnimaliaOrthopteraGryllidae

Ma, Qiao & Zhang, 2019

D2FCCDEE-2B74-5D59-8251-AF02DE19AE84

: 兰斗蟋 Figures 5 , 6 , 7 , 8B, E , 9B, E , 10B, E , 13B, E , 14C, D , Table 2

Velarifictorus (Velarifictorus) landrevus Ma, Qiao & Zhang, 2019: 104

##### Examined material.

5 males and 2 females, Yunnan, Mengla, Wangtianshu, 21°59.7'N, 101°58.8'E, X–12–23–2014, Zhang, Tao leg. The specimens were collected on grasses or leaf litter of a hillside.

##### Measurements.

**Male (*n* = 5)**: BL 19.49±0.60, HL 3.71±0.23, HW 6.52±0.31, PL 4.47±0.19, PW 6.20±0.23, FWL 9.32±0.98, MTL 5.42±0.22, HFL 13.82±0.26; **Female (*n* = 2)**: BL 18.59±0.09, HL 3.71±0.56, HW 5.81±0.24, PL 4.61±0.02, PW 6.34±0.08, FWL 4.72±0.14, HFL 13.93±0.23, OL 13.68±0.41.

##### Description.

**Male**: vertex broad and flattened, slightly inclined. Occiput slightly convex and almost as wide as pronotum. Frontal rostrum convex, inclined dorsally and ventrally, nearly1.5 times wider than antennal scape. Antennal scape shield-like. Ocelli transversely ovoid, almost equal in size. Eyes ovoid, about 1/5 length of head. Postclypeus shaped as narrow band, anteclypeus shaped as broad shield. Labrum shaped as rhombus, apical margin slightly round. End section of maxillary palpi almost as long as the third; end section of labial palpi depressed and widened, almost equal to the total length of remainder sections.

**Table 2.** Descriptive statistics of acoustic parameters in calling song samples of *Velarifictoruslandrevus*.

	*n*	minimum	maximum	mean	std
Chirp interval	26	2.629	5.191	3.910	1.281
Chirp duration	26	0.394	0.553	0.474	0.080
Chirp period	23	3.023	6.463	4.384	1.361
Chirp elements	23	9	15	12	3
Pulse interval	23	0.021	0.028	0.024	0.03
Pulse duration	23	0.011	0.024	0.017	0.006
Pulse period	23	0.032	0.052	0.042	0.010
Peak frequency	23	2.383	5.347	3.865	1.482

The time and frequency parameters are in seconds and kHz respectively. *n*= number of samples analyzed; std= standard deviation.

Pronotum broad and flattened. Median groove of pronotal disc distinct. Posterior margin straight and middle of anterior margin concave, and both margins almost equal in width.

Tegmina not reaching apex of abdomen. Chord veins two. Diagonal vein proximally bifurcated, one branch of connected to the proximal of chord vein, while another branch connected to CuA vein. Mirror shield-like, anterior margin and the posterior almost equal in width. Apical field about 1/5 length of basal field, possessing two or three wing cells.

Inner tympanum absent; external tympanum elongate-oval. Hind tibiae armed with dorsal spurs (numbered 6:6) almost equal in length; and apical spurs three outside (the dorsal one slightly longer than the dorsal spurs, the ventral and middle almost equal in length and about half length of the dorsal) and inner two (equal in length and about two times longer than the dorsal).

##### Genitalia.

Lateral lobes of epiphallus sheet-like, tapering, apically truncated, and pilose along margins. Middle lobe of epiphallus partially pentagon-like (dorsally viewed) and about half length of lateral lobes. Ectoparamere stripe-like, proximally broad, tapering, upward curved, and about twice as long as epiphallic lateral lobes.

##### Calling song.

**Figure 5.**
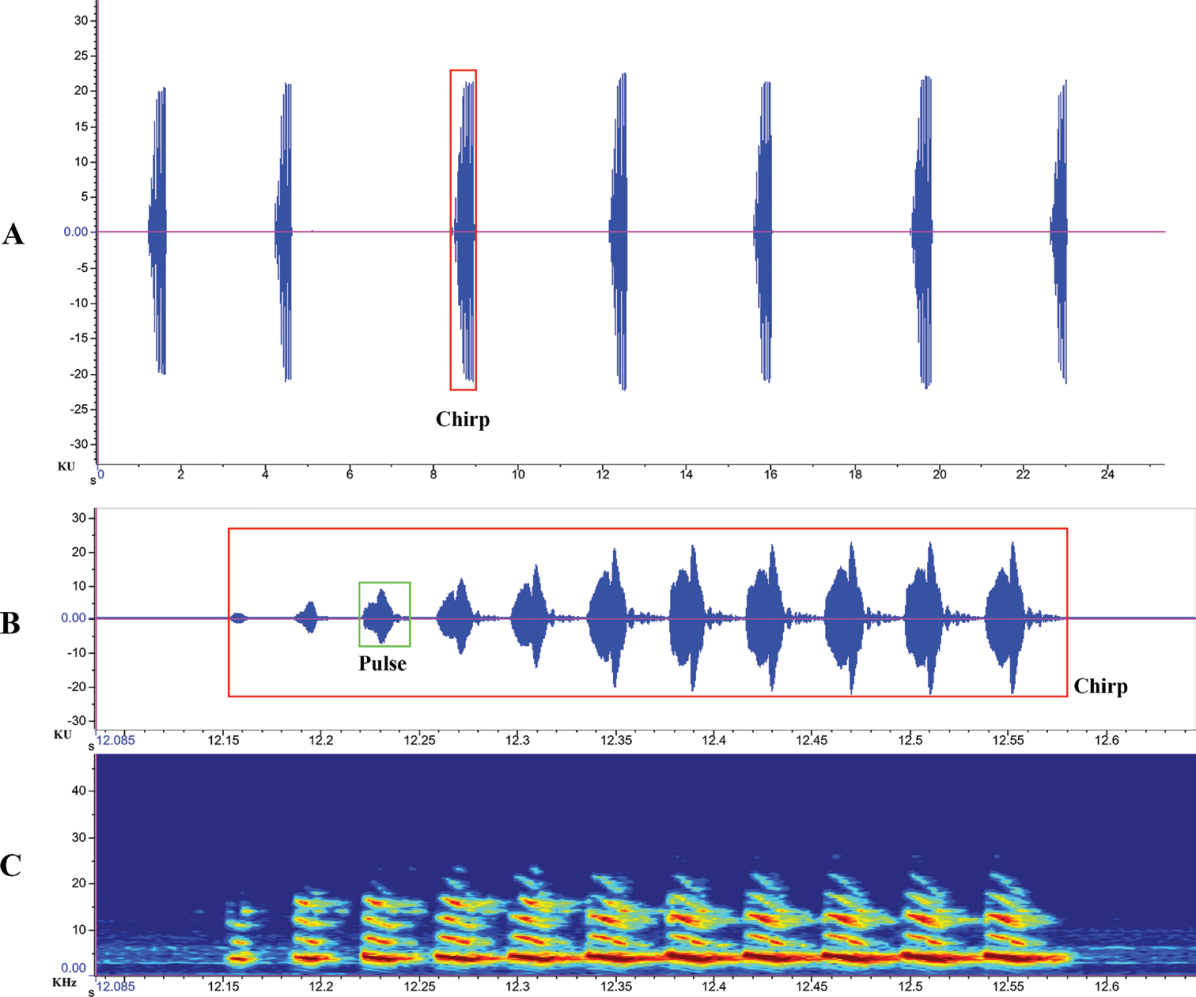
Calling song of *Velarifictoruslandrevus***A** oscillogram of chirp (amplitude versus time) **B** oscillogram of pulse (amplitude versus time) **C** spectrogram (frequency versus time).

Chirps lasting from 3.023 to 6.463 s (mean 4.384) and their duration varying from 0.394 to 0.553 s (mean 0.474). Each chirp equally contains 12 pulses (Fig. 5B). The peak frequency is between 2.383 and 5.347 kHz (mean 3.865) and the mean temperature is 27.8 °C. Regarding spectro-temporal variation, the frequency modulation is inclined downward (Fig. 5C).

**Figure 6.**
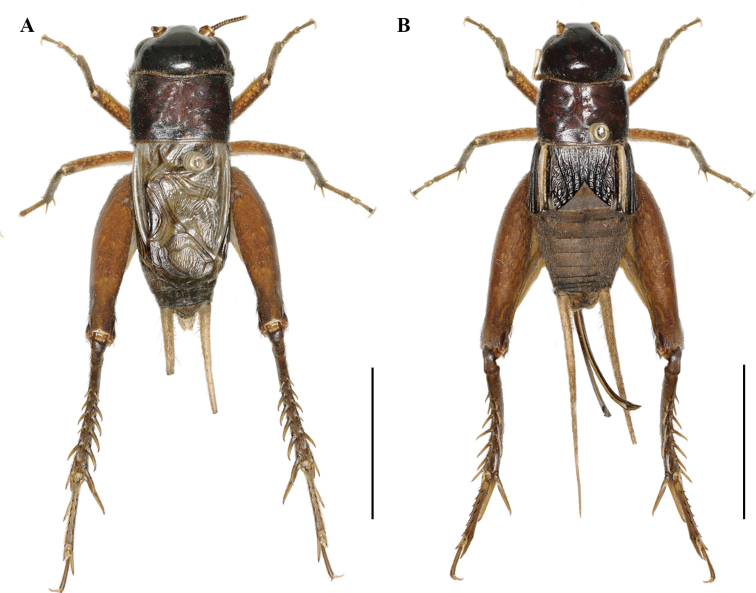
Bodies of *Velarifictoruslandrevus***A** male **B** female. Scale bars: 10 mm.

**Female.** Resembles the male. Tegmina reaches the apex of the second abdominal tergite (Fig. 6B) and is armed with longitudinal veins (inside shorter and progressively growing outward), dorsally four longitudinal veins, and laterally six Sc branches (the innermost of which medially and apically branched) (Fig. 13E). Ovipositor arrow-shaped, constricted, long, and with a somewhat sharp tip (Fig. 14 C, D).

##### Coloration.

Body chocolate brown. Head dark brown. End section of maxillary palpi light yellow. Cercus and legs yellowish brown. CuA and Sc of female ornamented with yellowish band.

**Figure 7.**
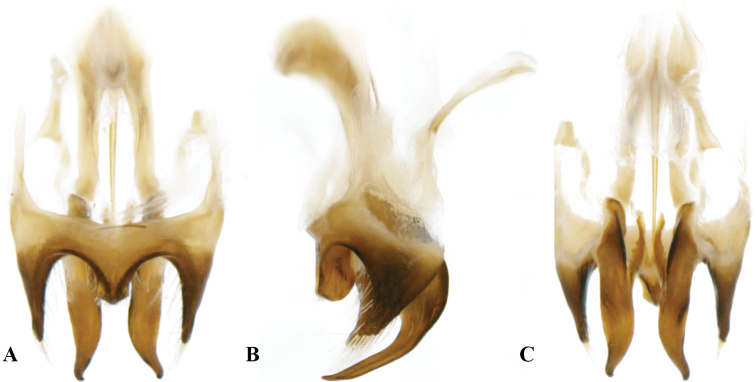
Genitalia of *Velarifictoruslandrevus***A** dorsal view **B** lateral view **C** ventral view.

**Figure 8.**
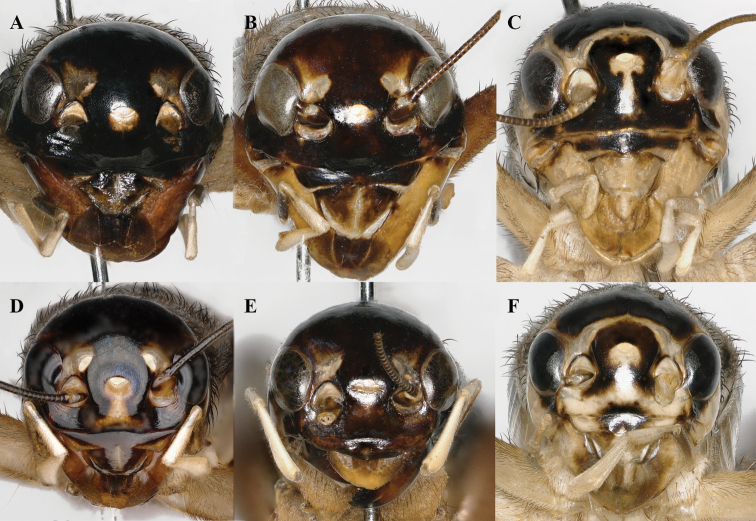
Front view of heads of *Velarifictorus***A, D***V.zhengi* sp. nov. **B, E***V.landrevus***C, F***V.flavifrons***A–C** male **D–F** female.

#### ﻿ Velarifictorus flavifrons

Taxon classificationAnimaliaOrthopteraGryllidae

Chopard, 1966

11023AEB-A105-5E21-AB84-67370A24EEA2

**Figure 9.**
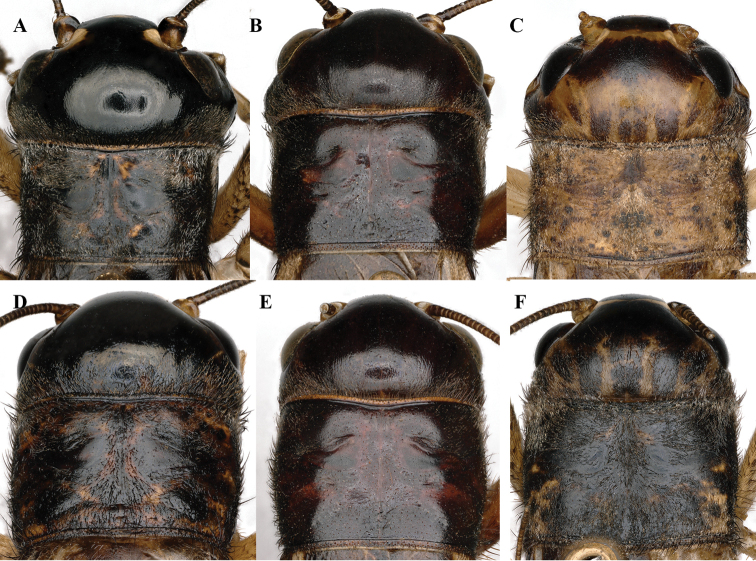
Dorsal view of heads and pronotum of *Velarifictorus***A, D***V.zhengi* sp. nov. **B, E***V.landrevus***C, F***V.flavifrons***A–C** male **D–F** female.

**Figure 10.**
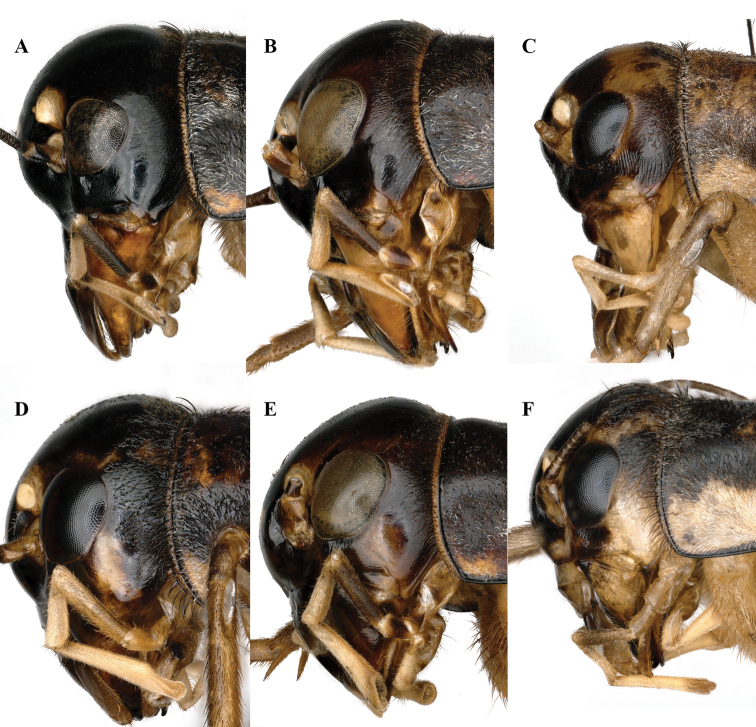
Lateral view of heads of *Velarifictorus***A, D***V.zhengi* sp. nov. **B, E***V.landrevus***C, F***V.flavifrons***A–C** male **D–F** female.

**Figure 11.**
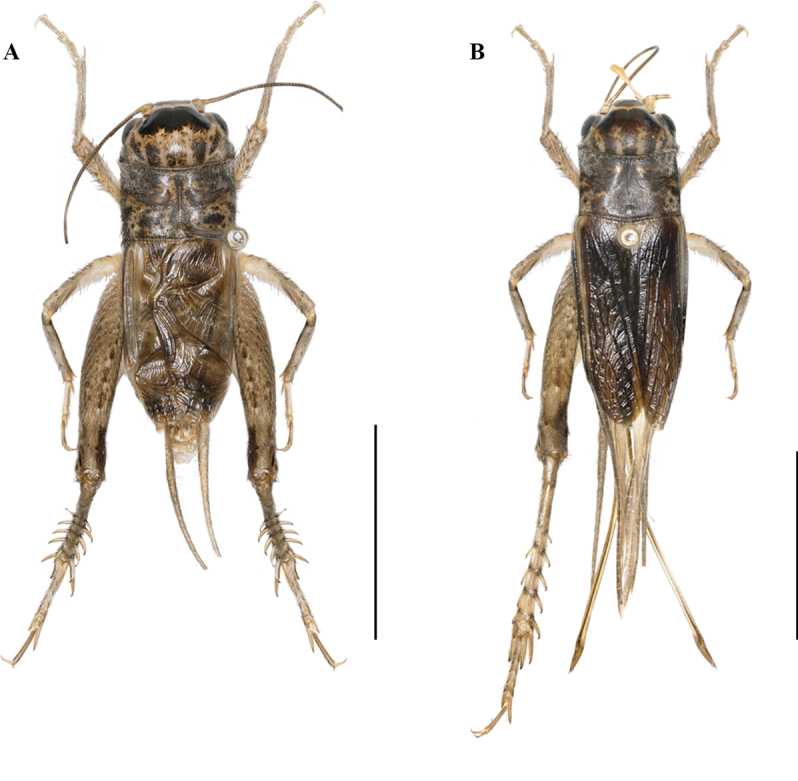
Bodies of *Velarifictorusflavifrons***A** male **B** female. Scale bars: 10 mm.

**Figure 12.**
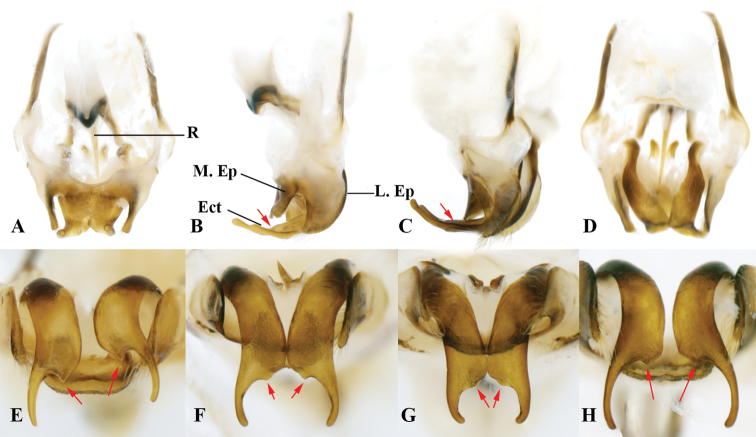
Genitalia of *Velarifictorusflavifrons***A** dorsal view **B, C** lateral view (ectoparamerea are movable and may touch or be separated from middle epiphallic lobe in some material) **D** ventral view **E–H** caudal view **E** inner branch observable and apically acute **F** inner branch small and asymmetry (observed five samples), as evident here, the left apically rounded and the right somewhat acute **G** inner branch relatively weak **H** inner branch similarly placed as in F but different in details.

**Figure 13.**
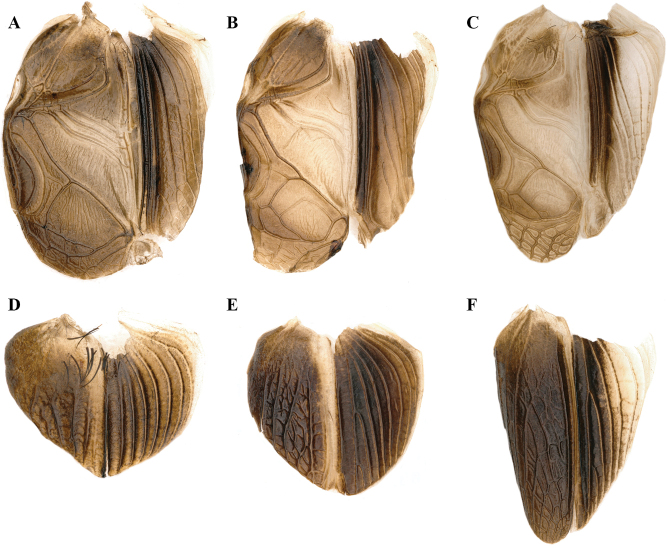
Right tegmina of *Velarifictorus***A, D***V.zhengi* sp. nov. **B, E***V.landrevus***C, F***V.flavifrons***A–C** male **D–F** female.

**Figure 14.**
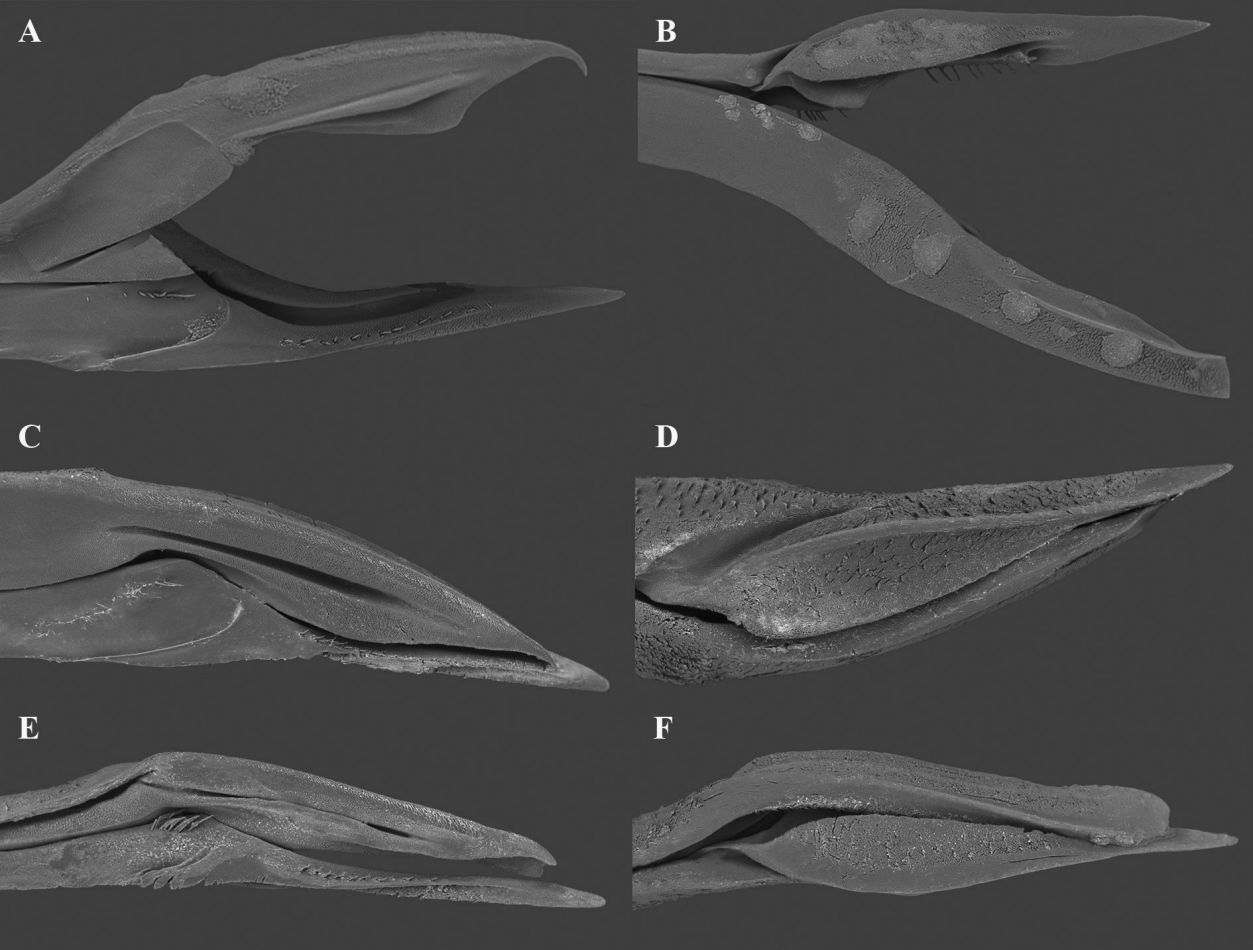
Ovipositor of *Velarifictorus***A, B***V.zhengi* sp. nov. **C, D***V.landrevus***E, F***V.flavifrons***A, C, E** inside of ovipositor **B, D, F** outside of ovipositor.

: 黄额斗蟋 Figures 8C, F , 9C, F , 10C, F , 11 , 12 , 13C, F , 14E, F

Velarifictorus flavifrons Chopard, 1966: 607; Chopard 1967: 124
Velarifictorus (Velarifictorus) flavifrons , Shishodia et al., 2010: 212; Kim and Hong 2014: 61; Zhang et al. 2017: 26; Chen et al. 2018: 502
Scapsipedus arorai Tandon & Shishodia, 1972: 281; synonymized by Vasanth 1993: 38
Scapsipedus bhadurii Bhowmik, 1967: 127; synonymized by Vasanth 1982: 123
Velarifictorus dehradunensis Tandon & Shishodia, 1974: 299; synonymized by Bhowmik 1985: 30
Velarifictorus jaintianus Biswas & Ghosh, 1975: 221; synonymized by Bhowmik 1985: 37
Scapsipedus lohitensis Tandon & Shishodia, 1972: 281; synonymized by Vasanth 1982: 123
Scapsipedus sikkimensis Bhowmik, 1967: 126; synonymized by Gorochov 2001:321
Velarifictorus yunnanensis Liu & Yin, 1993: 90, 93; synonymized by Gorochov 2001: 321

##### Examined material.

5 males and 2 females, Yunnan, Gongshan, 21°69.4'N, 101°57.3'E, Ⅵ–14–2019, Libin Ma leg.; 10 males and 2 females, Xizang, Motuo, Beibeng, 29°24.5'N, 95°17.6'E, Ⅴ–31–2019, Libin Ma leg.; 5 males, Guangxi, Jingxi, Longbang, 22°87.3'N, 106°32.8'E, Ⅴ–1–2019, Libin and Tao Zhang leg. Specimens in grass and leaf litter.

##### Measurements.

**Male (*n* = 15)**: BL 16.7±0.53, HL 3.23±0.23, HW 5.13±0.23, PL 3.13±0.76, PW 4.98±0.03, FWL 8.98±0.64, MTL 5.18±0.22, HFL 9.23±0.26; **Female (*n* = 4)**: BL 17.98±0.24, HL 3.87±0.54, HW 5.31±0.76, PL 4.43±0.03, PW 6.34±0.35, FWL 4.43±0.15, HFL 13.45±0.76, OL 13.63±0.18.

##### Description.

**Male**: vertex broad and flattened, slightly inclined. Occiput slightly convex, almost in equal length with pronotum. Frontal rostrum convex, inclined dorsally and ventrally, and nearly twice wider than antennal scape. Antennal scape shield-like. Median ocellus transversely ovoid, lateral ocelli ovoid. Eyes ovoid, about 1/3 length of head. Postclypeus shaped as narrow band, lower margin convex; anteclypeus trapezoidal. Labrum rhomboid, apical margin slightly rounded. End section of maxillary palpi slightly longer than the third; end section of labial palpi depressed and widened, almost equal to the total length of all other sections.

Pronotum broad and flattened. Median groove of pronotal disc distinct and ornamented with symmetrical blade-pattern on both sides. Posterior margin straight and middle of anterior margin concave inside, and both margins almost equal in width.

Oblique veins three. Diagonal vein proximally bifurcate, each branch connected to CuA vein. Chord veins three. Mirror shield-like, posterior margin slightly rounded, and dividing vein curved. The apical field almost as wide as mirror and armed with regular cells.

Inner tympanum relatively small or absent, and outer tympanum elongate-oval. Hind tibiae armed with dorsal spurs (numbered 5:6) almost equal in length; apical spurs external three (dorsal one about 3/2 length of dorsal spurs; ventral and middle nearly equal in length and almost equal to dorsal spurs) and inner two (equal in length and about 2/3 length of the dorsal).

##### Genitalia.

Lateral lobes of epiphallus sheet-like, apically rounded, and pilose. Middle lobe of epiphallus about 1/2 length of epiphallic lateral lobes, ventrally possessing projections and about 1/3 length of epiphallic lateral lobes. Ectoparamere proximally rod-like and distally sheet-like. Ectoparamere curved dorsally (degree of curvature variable). Apex of ectoparamere bifurcate, outer branch rather long (slightly longer than epiphallic lateral lobes) and apically rounded, and the inner relatively short (only a small protrusion and varied in size and shape: round or acute, or asymmetry with one round while another acute) (Fig. 12E–H).

##### Coloration.

Body brown. Occiput dark brown, vertex armed with six yellowish-brown strips. Anteclypeus dark brown, postclypeus light brown. With a broad yellowish-brown band between ocelli. End section of maxillary palpi dark brown and remainder sections light yellow.

**Female.** Resembles the male. Tegmen reaches apical abdominal tergite (Fig. 11B) and dorsally armed with irregular longitudinal veins and laterally with five Sc branches (innermost of which branches medially and apically) (Fig. 13F). Ovipositor brown, smooth, arrow-shaped, and slightly longer than posterior femur. The dorsal ovipositor valve apically truncated, whereas the ventral valve with apex slightly acute (Fig. 14E, F).

##### Remarks.

Gorochov (2001) considered *V.yunnanensis* to be a junior synonym of *V.flavifrons*. We checked the original descriptions and found that the figures of these species differ. To confirm the relationship of these species, we studied material collected from Yunnan Province (seven individuals), which should either bear the name of *V.flavifrons* or *V.yunnanensis*. Most of these specimens were collected at the same time and in the same location. We conclude that morphological variation of is present in the male genitalial complex (Fig. 12), as follows: (1) ectoparamere dorsally curved and variable in its curvature; (2) ectoparamere bifurcate and inner branch variable in size (clearly or weakly observable); (3) inner branch of ectoparamere morphologically variable (round or acute, or asymmetry with one acute and the other rounded). Thus, our observations confirm the synonymy of *V.flavifrons* and *V.yunnanensis* as proposed by Gorochov (2001).

## Supplementary Material

XML Treatment for Velarifictorus

XML Treatment for Velarifictorus zhengi

XML Treatment for Velarifictorus (Velarifictorus) landrevus

XML Treatment for Velarifictorus flavifrons
